# Evaluating the Diagnostic Accuracy of Reflectance Confocal Microscopy to Diagnose Skin Cancer: Protocol for a Prospective, Multicenter Study

**DOI:** 10.2196/resprot.9296

**Published:** 2018-08-09

**Authors:** Naomi D Herz, Anastasia Chalkidou, Fiona Reid, Stephen F Keevil, Andrew Coleman, Emma Craythorne, Rakesh Patalay

**Affiliations:** ^1^ King's Technology Evaluation Centre School of Biomedical Engineering & Imaging Sciences King's College London London United Kingdom; ^2^ Guy's and St Thomas' NHS Foundation Trust London United Kingdom

**Keywords:** melanoma, lentigo maligna, biopsy, dermoscopy, reflectance confocal microscopy

## Abstract

**Background:**

In the United Kingdom, 350,000 patients per year are referred to hospital clinics with suspicious moles, and approximately half undergo a biopsy to identify the 5%-10% who require further treatment. If cancer cannot be ruled out clinically and on the basis of biopsy results, the lesion is surgically removed. One type of precancerous mole, called lentigo maligna, is particularly challenging to delineate and treat. Reflectance confocal microscopy (VivaScope, Caliber Imaging & Diagnostics) is an imaging technique that can supplement dermoscopy in identifying whether a clinically suspicious mole is malignant and can better assess lentigo maligna margins for excision. It allows clinicians to visualize the skin lesion to a depth of 200 microns with subcellular resolution, described as quasi-histological, and therefore better guide more accurate diagnoses.

**Objective:**

The aim of this paper is to describe a prospective, single blinded, multicenter study to examine patients with clinically suspicious moles or lentigo maligna to determine whether confocal microscopy can both reduce the number of unnecessary biopsies of moles and more accurately guide the surgical excision margins of lentigo maligna.

**Methods:**

This study will prospectively recruit adults into the following two cohorts: diagnostic accuracy and margin delineation. The diagnostic accuracy cohort will assess people with clinically suspicious lesions suspected of being diagnosed with melanoma and having an equivocal finding on dermoscopy or persistent clinical suspicion despite normal dermoscopy. Diagnostic accuracy will include the sensitivity and specificity of VivaScope in comparison with the histological diagnosis as the gold standard for patients. The margin delineation cohort will assess the ability of VivaScope to accurately delineate the margins of lentigo maligna compared with that of dermoscopy alone using margins taken during Mohs micrographic surgery as the gold standard. The primary study outcomes will be the diagnostic accuracy of VivaScope for the first cohort of patients and margin agreement between VivaScope and the final pathology report for the second cohort of patients.

**Results:**

Funding for this proposed research is being secured.

**Conclusions:**

The outcomes of the proposed study will indicate how many biopsies of nonmelanoma lesions, which are potentially unnecessary, could be prevented. This would reduce patient anxiety and cost to the National Health Service (NHS) in the United Kingdom. Improved margin delineation of lentigo maligna could also improve the surgical clearance rates and decrease overall cost. The results would demonstrate whether the adoption of VivaScope would potentially benefit patients and the NHS.

**Registered Report Identifier:**

RR1-10.2196/9296

## Introduction

### Background

In the United Kingdom, 350,000 patients per year are referred to hospitals with suspicious moles, and approximately half undergo a biopsy to identify the 5%-10% who require further treatment [1]. Typically, a dermatologist diagnoses skin cancer based on clinical history and examination that is aided by a dermatoscope. If cancer cannot be ruled out, the lesion is surgically removed or in some cases, it is monitored by repeated visits to the clinic. Lentigo maligna (LM), a premalignant lesion that grows slowly on sun-exposed sites and can transform into a melanoma, is particularly challenging to treat. Because it is difficult to identify the transformation of LM into a melanoma, it is usually treated when it is found, before malignant transformation. Its margins are difficult to assess in a visual examination with or without the aid of a dermatoscope. The consequence of this uncertainty is that despite surgery with a 5 mm margin around the clinical edge, the treatment has high rates of incomplete excisions and recurrence rates are high [2].

Reflectance confocal microscopy (RCM) is a noninvasive real-time imaging technique used at the bedside. The VivaScope 1500 and 3000 (Caliber Imaging & Diagnostics) use near-infrared point-laser light to image the top layers of the skin, blood vessels, and pigment with a cellular resolution [3]. The technology can help identify whether a clinically suspicious mole is histologically suspicious and needs to be removed. It can also guide visualization of the true margins of LM prior to surgical removal. In these cases (as in this study), Mohs surgery can be used, which involves examination of the entirety of the surgical margins in multiple stages to ensure that the whole tumor is removed.

In 2015, the National Institute for Health and Care Excellence (NICE) Diagnostics Assessment Programme published Diagnostics Guidance 19 (DG19) [3], which examined the use of VivaScope and its potential role in the National Health Service (NHS). DG19 stated that there was insufficient evidence to recommend the routine adoption of VivaScope in the NHS for the assessment of skin cancer. The guidance identified a number of studies that have shown that VivaScope can improve the specificity of melanoma diagnosis when used as an adjunct to dermoscopy [3].

VivaScope examination in patients with difficult-to-diagnose moles has been shown to have higher specificity than dermoscopy [4]. This can potentially reduce unnecessary biopsies by enabling a more accurate clinical diagnosis.

VivaScope can also more accurately define the edges of LM in comparison with dermoscopy [5]. This is particularly important because these lesions occur on the face in the elderly and are often large, requiring complex reconstructive surgery. A more refined definition of LM margins is expected to better guide patients’ expectations of treatment, improve surgical planning and cure rates, and decrease the amount of normal tissue removed during surgery.

Although VivaScope may improve patient care and management [4-11], there is a lack of data from the United Kingdom [3]. It is felt that the applicability of the existing evidence to a UK population is unclear. Relevant differences between the United Kingdom and other countries with evidence of the impact of VivaScope are as follows: the underlying incidence of melanoma in different patient populations, the fact that most treatment in United Kingdom is performed by a public health system (NHS), there are fewer dermatologists in the United Kingdom, and most screening in the United Kingdom occurs in primary care prior to specialist referral. Therefore, the guidance recommended further research to address uncertainties in the potential benefits of using VivaScope to patients and the NHS.

The proposed diagnostic accuracy study will (1) assess the specificity, sensitivity, and positive and negative predictive value of VivaScope (following dermoscopy) to diagnose melanoma using the histological assessment of the surgically excised lesion as the gold standard and (2) assess the ability of VivaScope to accurately delineate the pathological margins of LM compared with that of dermoscopy alone using histological assessment of the lateral margins taken during Mohs micrographic surgery as the gold standard. If the outcomes of the proposed study are in favor of VivaScope, its adoption would potentially benefit patients and the NHS.

### Objectives

The aim of this study is two-fold; the study aims (1) to assess the efficacy of VivaScope as an additional diagnostic tool prior to the surgical management of patients with suspected melanoma (first cohort) and (2) to define the surgical margins before the surgical management of LM (second cohort).

## Methods

### Type of Study

This is a prospective, experimental, multicenter study with a diagnostic accuracy cohort and a margin delineation cohort. The study design was chosen because it is pragmatic and represents the current NHS pathway for the diagnosis of melanoma.

### Setting

For a UK study, patient recruitment for the diagnostic accuracy cohort should occur in skin cancer screening clinics within the NHS. Image interpretation should also be carried out at additional reporting sites. The sites can be linked to the image interpretation sites with VivaNet, a Web-based portal for image viewing and reporting provided by the manufacturer of VivaScope.

Recruitment to the margin delineation cohort must be from a Mohs center in the United Kingdom. The involvement of additional independent reporting clinicians for the margin delineation protocol is not possible because the handheld VivaScope 3000 is used to delineate the margin using the real-time images.

### Study Population

Consecutive patients will be included if they are at least 18 years of age and can give written informed consent.

#### Diagnostic Accuracy

The diagnostic accuracy cohort will include patients with pigmented lesions suspected of being diagnosed with melanoma and having an equivocal finding on dermoscopy or persistent clinical suspicion (despite normal dermoscopy), who are undergoing excision or being followed up for monitoring. In addition, patients with equivocal lesions that are being monitored will also be imaged but will not have a histologically confirmed diagnosis. Such patients will be reviewed at 3 months and followed up at 1 year. If the lesion has not been removed by 1 year, this will be used as a surrogate marker for true negatives. Potentially, lesions considered equivocal on dermoscopy and initially intended for monitoring, but subsequently considered suspicious of melanoma on confocal microscopy, could be considered for excision. It will be documented if this influenced the decision to excise the lesion at this time. Where a patient has more than one eligible lesion, a maximum of one lesion per patient will be assessed. In these cases, the clinician will be instructed to include the most suspicious lesion in the study. Assessment will done by an experienced dermatologist.

Patients will be excluded from the diagnostic accuracy cohort if they have a clear positive finding (melanoma) from dermoscopy, as assessed by an experienced dermatologist or have a clear negative finding (no melanoma) from dermoscopy, as assessed by an experienced dermatologist, unless there is persistent clinical suspicion. Patients who have atypical mole syndrome or a genetic disease with high risk of melanoma (eg, xeroderma pigmentosum), hyperkeratotic lesions, and lesions within mucous membranes (eg, inside the mouth, very close to the eyes, and on the genitals), where it is not possible to perform imaging, will also be excluded.

Patients are assessed for inclusion or exclusion based on the clinical examination of the mole (usually includes a clinical history, unaided visual and dermoscopic assessment of the mole, and assessment of a patient’s other moles for comparison). The assessment by the referring clinician that a mole is equivocal is made primarily on the basis of dermoscopy findings but other aspects of the assessment will also influence this. The dermoscopic assessment of the lesion being equivocal by a referring clinician will be based on a combination of algorithmic and intuitive approaches. This may result in a mole being deemed equivocal despite a normal formal dermoscopic assessment score. Because the inclusion criteria for this study are designed to reflect the clinician’s suspicion and normal clinical practice, the referring clinician will not be expected to formally document a dermoscopic score, only to indicate the reason for referral (ie, monitoring or excision). On entering the study, the patient will be assessed via a second dermoscopy examination by the research team. This examination will be formally scored and the dermoscopic image photographed. The patient will remain in the study despite the outcome of the study dermoscopy for the reasons explained above.

**Figure 1:**
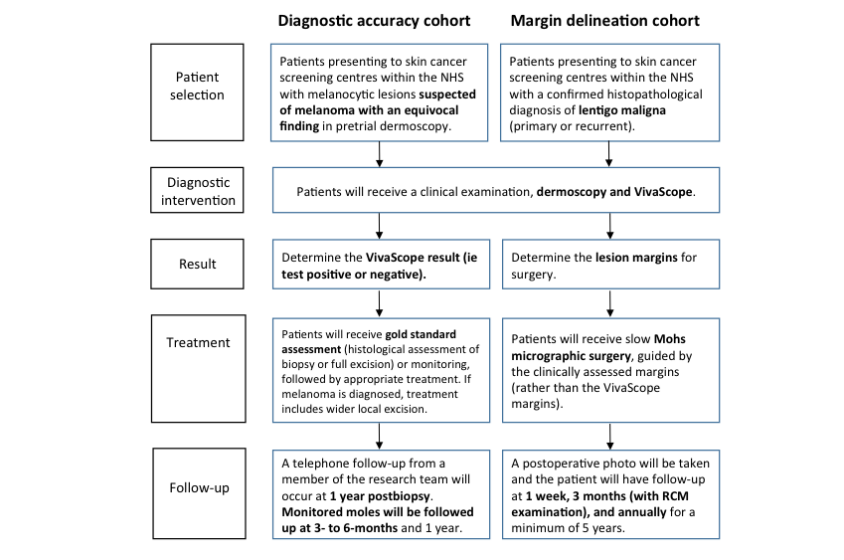
Study procedure. NHS: National Health Service; RCM: reflectance confocal microscopy.

#### Margin Delineation

The margin delineation cohort will include patients with a confirmed histopathological diagnosis of LM (primary or recurrent) who are undergoing Mohs surgery. Patients will be excluded if LM melanoma is diagnosed during the Mohs procedure.

### Study Procedure

The primary study outcomes will be the diagnostic accuracy of VivaScope for the first cohort of patients and margin agreement between VivaScope and the final pathology report for the second cohort of patients.

The study procedure is outlined in Figure 1.

#### Diagnostic Accuracy

In the diagnostic accuracy cohort, after the participant is enrolled and provides consent, VivaScope imaging will be used as an adjunct to clinical examination for all equivocal moles. The clinical examination will take approximately 5 minutes with the dermatoscope and approximately 15 minutes with VivaScope 1500 (fixed head). The investigating clinicians will assess the moles as safe, equivocal (either monitor or excise), or suspicious. Based on current practice, moles that are monitored are followed up at 3 months to assess changes over time. At this appointment, a decision is usually made to either excise the mole or discharge the patient. Lesions considered equivocal on dermoscopy but suspicious of melanoma on confocal microscopy may be reclassified for excision, in which case this would be documented.

A medical photograph, including dermoscopic images of the lesion, will be taken prior to surgical excision or monitoring of the lesion. To avoid potential bias, the dermoscopy score of the lesion assessed by the research team, medical photograph, type of clinical concern, clinical history, and patient risk factors for melanoma will all be assessed and documented before any histological results are available and made known to the investigator. For patients with multiple lesions, the most suspicious equivocal lesion will be imaged.

#### Margin Delineation

For the margin delineation cohort, once the participant is enrolled and consent is obtained, relevant clinical history and a preoperative photograph will be taken. The clinical margins of LM will be determined by clinical and dermoscopic examination, which will take approximately 15 minutes. A trained professional will also examine the lesion margins with the handheld VivaScope 3000 device, which will take approximately 45 minutes. The aim will be to delineate the tumor circumferentially, imaging radially outwards from the center in 4-8 directions. Where no features of LM are seen, a mark will be placed on the skin. A photograph of the marks will be taken with a ruler placed on the skin for scale and with this information, the preoperative size can be calculated via computer software.

Prior to surgery, the margin will be delineated clinically and then using VivaScope. These margins will both be photographed so that any differences can be calculated. Margin assessment may be performed during the surgical preassessment stage and not on the day of surgery, so as not to delay surgery on the day. For cases not delineated by both techniques on the day, the clinical margin will be redrawn and photographed again. Because LM grows slowly, we do not expect any differences in the measured clinical size of LM (between the preoperative assessment and on the day of surgery). Any differences seen will be assumed to be attributable to variation in the measuring technique, unless photography shows an unequivocal change in the size and shape of LM.

All patients going through surgery will undergo slow Mohs micrographic surgery. The margins, as assessed by visual examination (including dermoscopy), will be used to guide the first stage of surgery rather than the RCM-measured margin. The first stage of slow Mohs surgery will be performed with a 2-mm margin, in addition to the clinical margin. The excised tissue will be fixed in formalin and then sectioned and stained using hematoxylin and eosin stains the following day. All slides will be read by a Mohs surgeon and a histopathologist. During each further stage of Mohs surgery, 2 mm of tissue around the positive margin will be removed.

Once the lesion is histologically clear, the number of layers (number of small pieces of skin removed during Mohs surgery) and their locations (taken from photographs) can be used to calculate the increase in size from the clinical margin. This will be aided by the digital measurements obtained from clinical photography to determine the surgical defect size, and this method may account for skin tension (which is lost once the skin is cut and changes in size and color). Therefore, for example, if the clinical margin were correct initially, the surgical defect size would be measured to be exactly 2 mm larger in all directions. This same approach will be used to compare the clinically and confocally delineated margins. The difference between the presurgical margins previously measured using VivaScope and the calculated margins of the surgical defect will be calculated.

LM patients will have follow-up at 3 months and annually for a minimum of 5 years to monitor recurrence.

### Outcome Assessment and Data Collection

#### Diagnostic Accuracy

Clinical assessment of the lesion and confocal imaging will be performed prior to surgical excision. It will be documented if VivaScope imaging findings are positive, negative, or the lesion could not be imaged (due to technical reasons). As noted previously, it will also be documented if the imaging resulted in an equivocal mole being reclassified as a suspicious melanoma, affecting the decision to excise the lesion. Additional remote clinicians will also perform an assessment independently. These remote assessing clinicians will have access to the relevant clinical history, dermoscopy, and clinical and confocal images. However, they will be blinded to the histological diagnosis. The clinicians will be asked to complete a Web-based evaluation form that will include a description of VivaScope imaging findings as well as a diagnostic judgment rated as positive or negative for the presence of cancer. VivaNet, a telepathology network for reviewing VivaScope imaging (not in real time), will be used to assess the images remotely.

Histopathological assessment, the reference standard for diagnostic accuracy, will be performed by a consultant dermatopathologist blinded to the result of RCM examination to eliminate review bias.

#### Margin Delineation

For the second cohort, the size of the margins will be recorded for VivaScope measurement as well as for slow Mohs surgery. The calculation of the surgical defect will also be recorded. The outcome measure will be the difference between the predicted margins using VivaScope and the Mohs size of the defect in the same patient. Thus, we are comparing the confocal microscope margin with the clinically predicted margin in each patient. Additional data will be collected on the time it takes to obtain test results, interobserver variability in the interpretation of VivaScope imaging, imaging failure rate, morbidity associated with biopsy or surgery, and adverse events from biopsy or surgery. The clinician administering the tests will record results on data collection forms by hand.

### Sample Size

#### Diagnostic Accuracy

Based on a systematic review and meta-analysis, the expected VivaScope specificity compared with the gold standard of histopathology is 90% [4], but for this calculation, a conservative estimate of 80% was used. A sample of 100 (true negatives) would provide a 95% CI for this VivaScope specificity of 70.8%-87.3%. This would allow us to conclude that the true specificity is >70%.

Assuming a disease prevalence of 20% (among this patient group who are equivocal on dermoscopy), 80% of patients tested will be true negatives on biopsy or after 1 year of monitoring without excision. Therefore, to achieve 100 true negatives, 125 patients will be included in this study (ie, tested by VivaScope, dermoscopy, and biopsy or monitoring).

#### Margin Delineation

The actual surgical margins achieved (which will have been guided by dermoscopy) will be compared with the hypothetical margins indicated by VivaScope for each patient. The paired mean difference in margins between surgery and VivaScope will be compared using the paired *t* test. Assuming a moderate standardized effect size of 0.4 and 90% power, a sample of 68 patients is required (PASS v15.0 software; NCSS, Utah, USA). A standardized effect size has been used owing to the absence of any known SD for paired differences in margins between these two approaches.

### Statistical Analyses

#### Diagnostic Accuracy

We will calculate the specificity of VivaScope (95% CI) in predicting true negatives, as defined by histopathology or 1 year of monitoring without excision of the lesion. Specificity is calculated as negative VivaScope results divided by true negatives. The sensitivity of VivaScope will also be calculated, as positive VivaScope results in the numerator divided by true positives in the denominator, to check that the expected gain in specificity is not at the cost of sensitivity. True positives will be defined by the histopathology of excised lesions, including those excised during the 1-year monitoring period. Positive and negative predictive values will also be calculated.

We will compute kappa statistics to calculate the interobserver agreement. The conventional classification on the basis of kappa statistics from almost perfect agreement (>.81), moderate agreement (.41-.60), and slight to poor agreement (<.20) will be used [12].

#### Margin Delineation

McNemar’s test will be used to compare the frequency of surgery documenting clear margins for VivaScope compared with clinical examination with dermoscopy, and the paired *t* test will be used to compare the mean margins.

### Ethics and Governance

Prior to initiating this study, the protocol would need Health Research Authority (HRA) approval, wherein HRA staff and an independent Research Ethics Committee will assess governance and ethical compliance for projects led from the NHS in the United Kingdom. Patients in the diagnostic accuracy cohort will be approached after their initial appointment if they are eligible to participate, and LM patients will be approached during their first consultation. Patients will be given information about the study and asked whether they would like to participate. If the patient is interested, the clinician will explain the aims, methods, and anticipated benefits of the study. The patient will be able to ask any questions or voice any concerns about the study prior to giving written consent. The patient will be able to withdraw from the study at any time.

The investigator has a responsibility to ensure that patient anonymity is protected and maintained. The investigator will ensure that identities are protected from any unauthorized parties. Information regarding study patients will be kept confidential and managed in accordance with the General Data Protection Regulation, NHS Caldicott Guardian requirements, The Research Governance Framework for Health and Social Care, and Research Ethics Committee approval. A pseudoanonymized identifier will be necessary for the margin delineation cohort.

Data will stay in the NHS and within the University sponsor’s computer system under normal arrangements for patient confidentiality and will include encryption and locked storage of all patient data. The chief investigator, principal investigator, and authorized researchers on the study team will have access to the information for purposes of data monitoring, validation, and analyses.

## Results

Funding for this proposed research is being secured.

## Discussion

### Strengths

This study aims to address the uncertainties identified in NICE DG19 by carrying out a prospective diagnostic accuracy study in the United Kingdom. NICE DG19 states that additional evidence should be collected relating to the use of VivaScope to inform decisions as to whether to biopsy and excise suspicious skin lesions as well as to define margins in LM patients.

The implementation of VivaScope in clinical practice has the potential to improve the accuracy of the diagnostic process. VivaScope is noninvasive and would reduce the number of surgical interventions. It would also lead to a better patient experience, particularly lessening patient anxiety due to uncertainty while waiting for a diagnosis. In the margin delineation cohort, VivaScope could minimize the amount of normal tissue excised unnecessarily and reduce the duration and number of stages of the Mohs surgery.

The outcomes captured in the study aim to address the recommendations from NICE DG19, which prompted the development of this protocol. NICE will update its guidance for clinical use if substantive evidence is generated as part of this study on VivaScope. NICE guidance would give a strong steer to the national and international adoption of VivaScope. Thus, this study would have a high impact.

### Challenges

Extending the study to multiple sites may be a better demonstration of applicability to the NHS. A major current drawback to this is the lack of suitable clinical centers in the United Kingdom that currently have the expertise in using VivaScope. In practice, the variable levels of expertise and cost of training could be a barrier for the adoption of the device. There is potential to find greater benefit in clinics with a large cohort of patients being monitored for high-risk moles but this would apply to only a limited number of services in the United Kingdom, as opposed to every hospital that has referrals for skin cancer.

### Future Directions

This study should influence a wider uptake of VivaScope for the diagnosis of pigmented lesions at the bedside, reducing unnecessary excisions. The results from this study should also increase the use of VivaScope for margin delineation in LM patients in the United Kingdom. As the technique becomes more established, it will be useful to conduct further research on the use of VivaScope to replace confirmatory biopsies for basal cell carcinomas prior to definitive treatment.

## Abbreviations

DG19: Diagnostics Guidance 19
LM: lentigo maligna
NHS: National Health Service
NICE: National Institute for Health and Care Excellence
RCM: reflectance confocal microscopy
